# The Role of Endothelial Dysfunction in Fracture Healing: Mechanisms and Potential Effects on Skeletal Repair

**DOI:** 10.3390/ijms27167278

**Published:** 2026-08-14

**Authors:** Jakub Michalczak, Jacob Znamierowski, Justin Bondarowicz, Wiktoria Małgorzata Zgoda, Mateusz Michalczak, Anne Prigent-Tessier, Christelle Basset, Tomasz Tokarek

**Affiliations:** 1 Center for Innovative Medical Education, Jagiellonian University Medical College, 30-688 Krakow, Poland; jakub.michalczak@student.uj.edu.pl (J.M.); jacob.znamierowski@student.uj.edu.pl (J.Z.); justin.bondarowicz@student.uj.edu.pl (J.B.); wiktoria.zgoda@student.uj.edu.pl (W.M.Z.);; 2 CAPS UMR 1093, Institut National de la Santé et de la Recherche Médicale, Université Bourgogne Europe, 21000 Dijon, Francechristelle.basset@u-bourgogne.fr (C.B.); 3 Center for Invasive Cardiology, Electrotherapy and Angiology, 33-300 Nowy Sacz, Poland

**Keywords:** fracture healing, endothelial dysfunction (ED), angiogenesis, bone regeneration, cardiovascular disease, nonunion, microvascular function

## Abstract

Fracture healing depends on coordinated osteogenesis and restoration of the vascular microenvironment. Endothelial cells support skeletal repair through angiogenesis, tissue perfusion and angiocrine signaling that regulates osteoprogenitor recruitment and differentiation. This narrative review examines endothelial dysfunction (ED) as a potential systemic contributor to impaired fracture healing by integrating evidence from vascular biology, experimental models and clinical studies. ED is characterized by reduced nitric oxide (NO) bioavailability, oxidative stress, inflammation and impaired vascular repair. These changes may disrupt angiogenic–osteogenic coupling through altered hypoxia-inducible factor 1-alpha subunit (HIF-1α)/vascular endothelial growth factor (VEGF) signaling, endothelial Notch activity, platelet-derived growth factor (PDGF)-mediated vascular remodeling and endothelial progenitor cell (EPC) mobilization. Conditions associated with endothelial dysfunction, including diabetes, aging, chronic kidney disease (CKD), smoking, obesity and chronic inflammatory disease, are also linked to delayed union, nonunion and poorer orthopedic outcomes. Cardiovascular disease and perioperative cardiovascular instability may further impair perfusion and physiological reserve during repair. However, the available evidence is predominantly experimental or observational, and direct causal evidence in fracture patients remains limited. Prospective studies combining standardized endothelial assessments with fracture-healing outcomes are needed to clarify clinical relevance and identify potential therapeutic targets.

## 1. Introduction

Fracture repair is a dynamic and tightly regulated process that recapitulates key aspects of skeletal development. It progresses through overlapping phases of inflammation, repair, and remodeling, requiring coordinated interactions between cellular responses, molecular signaling, and mechanical stability [1,2,3]. Disruption at any stage can result in delayed union or nonunion, reflecting the complexity of skeletal regeneration.

Adequate vascular supply is essential for successful fracture healing. Early angiogenesis restores the disrupted microcirculation, enabling oxygen and nutrient delivery necessary for callus formation and maturation [4,5]. Beyond this supportive role, endothelial cells actively regulate bone repair through the release of angiocrine signals that influence osteogenic cell recruitment and differentiation [6]. These observations highlight the vasculature as a critical regulator of skeletal regeneration rather than a passive conduit for perfusion.

**Figure 1:**
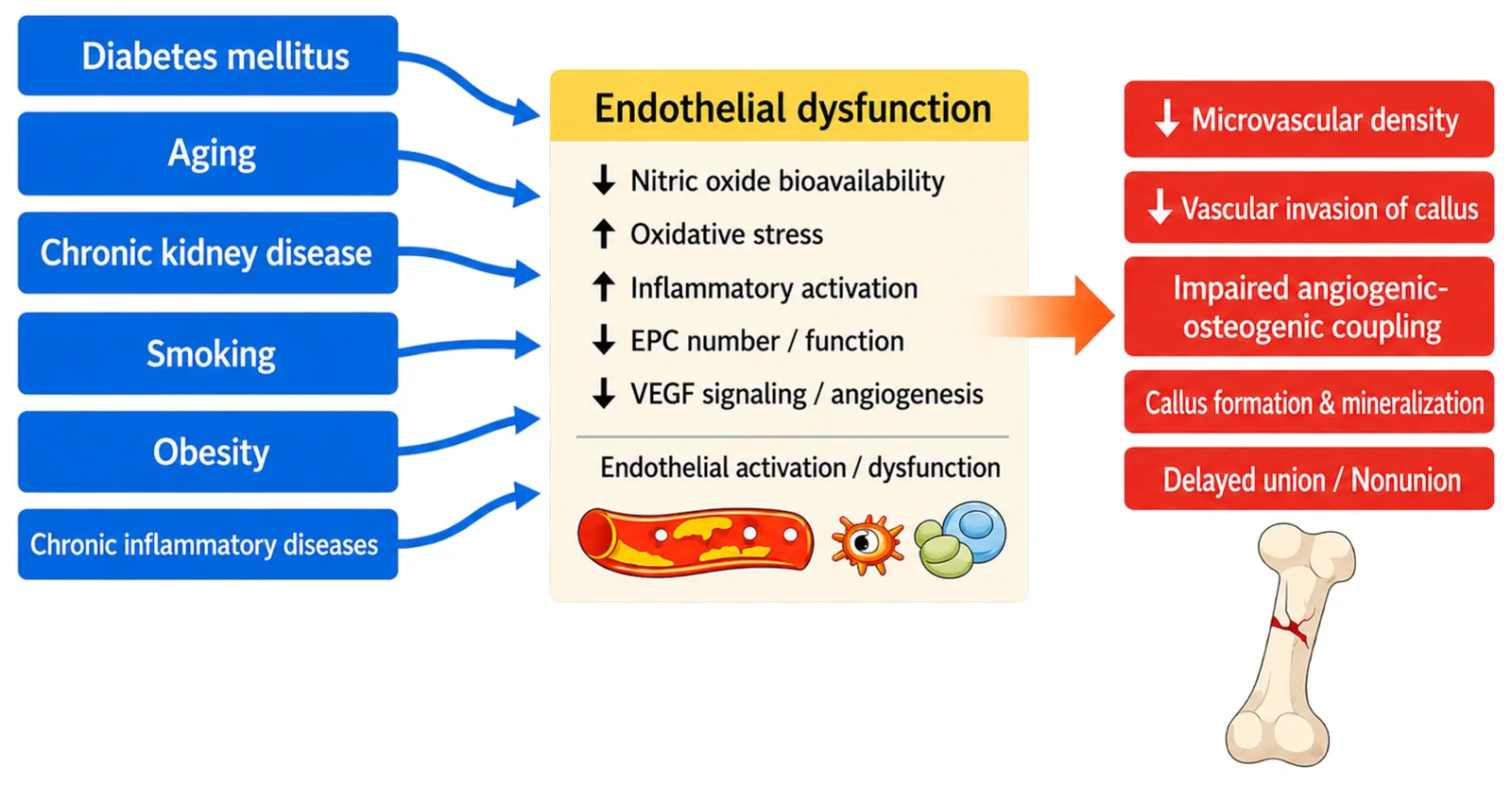
Endothelial dysfunction links systemic disease to impaired fracture healing.

Despite advances in surgical management, impaired fracture healing remains a significant clinical problem associated with substantial morbidity and socioeconomic burden [7,8]. The increasing prevalence of aging and cardiometabolic disease have further amplified this challenge, consistently linked to poorer skeletal outcomes, including both impaired fracture healing [9,10] and increased fracture risk [11]. These trends underscore the importance of systemic biological factors that may compromise regenerative capacity (Figure 1).

Although vascular processes are increasingly recognized in fracture biology, most research has focused on osteogenic mechanisms, and the contribution of systemic endothelial dysfunction (ED) remains incompletely defined. We synthesize mechanistic and clinical evidence that systemic ED may impair angiogenic–osteogenic coupling and contribute to delayed union and nonunion. By integrating insights from vascular biology, bone regeneration, and clinical studies, this review provides a framework for understanding how vascular pathology may influence skeletal repair.

For clarity, we distinguish direct fracture-healing outcomes (delayed union, nonunion, time to union, callus formation, and biomechanical/radiographic measures of repair) from related but distinct outcomes discussed here only as supporting context: fracture risk or incidence (e.g., BMD, FRAX score, fragility fracture prevalence), post-fracture mortality, and post-fracture functional recovery. Evidence of the latter types is identified explicitly as such and is not treated as direct evidence of impaired fracture repair.

### Literature Search

This article is a narrative review and is not a systematic review. Accordingly, it was not registered in a systematic review protocol database. The search approach is reported below for transparency and to indicate the scope of the literature considered.

The literature was identified primarily through PubMed searches combining the following concepts. Search terms combined vascular and skeletal concepts, including (“endothelial dysfunction” OR “endothelial function” OR “flow-mediated dilation” OR “endothelial progenitor cell” OR angiogenesis OR “type H vessel” OR “nitric oxide”) AND (“fracture healing” OR nonunion OR “delayed union” OR “bone regeneration” OR callus OR osteogenesis). Reference lists of retrieved articles and relevant reviews were hand-searched to identify additional primary studies, and landmark primary studies were prioritized over secondary reviews for mechanistic claims.

Articles were considered if they addressed endothelial or microvascular biology in bone, fracture-healing outcomes, or systemic conditions linking the two. Studies of bone metastasis, primary bone tumors, or implant-related osteolysis without a fracture-repair endpoint were not included. Because the topic spans vascular biology and orthopedic trauma fields with limited methodological overlap, article selection was necessarily interpretive rather than exhaustive, and we indicate throughout where evidence is direct rather than inferential.

## 2. Endothelial Dysfunction: Basic Concepts and Clinical Importance

The vascular endothelium is a dynamic, metabolically active organ that regulates vascular tone, permeability, inflammation, and thrombosis. Through the release of vasoactive mediators such as nitric oxide and endothelin-1, endothelial cells maintain perfusion and vascular homeostasis, while also coordinating immune cell trafficking and tissue repair processes [12,13,14].

ED refers to a pathological state characterized by impaired vasomotor regulation, reduced nitric oxide bioavailability, and a shift toward a pro-inflammatory and pro-thrombotic phenotype. This condition is increasingly recognized as a systemic disorder affecting multiple vascular beds simultaneously [15]. Mechanistically, oxidative stress plays a central role by reducing nitric oxide availability and promoting inflammatory signaling, leading to impaired perfusion and sustained endothelial activation [16,17].

We propose that microvascular dysfunction, rather than large-vessel disease, may represent the dominant determinant of tissue perfusion and angiogenesis in this context, given the callus’s dependence on microvascular ingrowth. Endothelial heterogeneity further shapes vascular function across tissues. While large-vessel endothelial cells primarily regulate blood flow, microvascular endothelial cells are specialized for oxygen delivery, nutrient exchange, and paracrine signaling [18,19,20,21]. Within bone, these microvascular networks actively regulate skeletal homeostasis through angiocrine signaling that supports osteoblast differentiation and coordinates angiogenesis with osteogenesis [20]. Disruption of these interactions may impair callus formation and mineralization, providing a possible link to delayed union and nonunion [21,22].

Clinically, ED represents a central link between cardiometabolic risk factors including diabetes, hypertension, dyslipidaemia, and smoking and vascular disease [23]. These same conditions are also associated with impaired fracture healing, supporting the concept that systemic vascular dysfunction may influence skeletal regeneration.

### Endothelial Function Assessment in Research

Endothelial function can be evaluated using both functional vascular testing and circulating biomarkers. Non-invasive methods such as flow-mediated dilation (FMD), nitroglycerin-mediated dilation (NID), and reactive hyperaemia index (RHI) assess different aspects of vascular reactivity. FMD primarily reflects endothelium-dependent vasodilation, NID provides an endothelium-independent comparison, and RHI assesses peripheral reactive hyperaemia and microvascular function [24].

Circulating biomarkers provide complementary information by reflecting systemic endothelial activation or injury. These include soluble adhesion molecules Vascular Cell Adhesion Molecule 1 (VCAM-1), intercellular adhesion molecule 1 (ICAM-1), Endothelial-selectin (E-selectin), asymmetric dimethylarginine (ADMA) as an inhibitor of nitric oxide signaling, and von Willebrand factor as a marker of endothelial damage [25]. Unlike localized functional testing, these markers capture global vascular status, which may be particularly relevant in conditions characterized by diffuse ED. However, variability in biomarker levels and the lack of standardized thresholds currently limit their direct clinical application in disease populations.

Throughout this review, we distinguish three related but non-equivalent constructs: (1) systemic ED, measured directly via flow-mediated dilation or circulating biomarkers of whole-body vascular status; (2) local bone/callus microvascular dysfunction, impaired perfusion or endothelial signaling within the healing fracture itself; and (3) reduced angiogenesis or microvascular density, a structural endpoint that can result from either unrelated causes such as mechanical disruption or hypoxia alone. Much of the clinical literature linking systemic disease to impaired healing establishes disease-level associations with each construct independently, rather than measuring endothelial function and fracture outcome in the same patients; we note this distinction explicitly where it applies.

## 3. Vascular Aspects of Bone Healing

Fracture healing occurs through two biological pathways: primary (direct) and secondary (indirect) repair, each defined by mechanical stability and vascular dynamics. Direct healing requires absolute stability and preserved microcirculation, allowing intracortical remodeling without callus formation. In contrast, indirect healing, by far the predominant clinical pattern, involves inflammation, callus formation, and robust angiogenesis, making it particularly dependent on vascular regeneration and systemic biological factors [1,3].

### 3.1. Direct Healing

Direct fracture healing occurs under conditions of absolute stability and relies on a more limited, tightly localized vascular process than the extensive neovascularization required for indirect healing. Repair proceeds through cutting cones, in which osteoclasts resorb bone at the leading edge while a trailing capillary loop and osteoblasts restore lamellar structure across the fracture interface [3]. Because this process still depends on an intact, advancing microvascular supply, we propose that vascular compromise or endothelial dysfunction could, in principle, impair healing even when mechanical fixation is optimal, though this has not been directly studied in the context of direct fracture healing.

### 3.2. Indirect Healing

Indirect healing, the predominant mechanism of fracture repair, occurs when relative stability permits callus formation. Fracture-induced vascular disruption leads to hematoma formation and a hypoxic microenvironment that initiates inflammation and angiogenic signaling. Early recruitment of immune cells, particularly macrophages, drives the release of cytokines and growth factors, including vascular endothelial growth factor (VEGF), linking inflammation to neovascularization [26,27]. During the reparative phase, mesenchymal progenitor cells differentiate into chondrocytes and osteoblasts, forming a fibrocartilaginous callus that is progressively replaced by woven bone. Vascular invasion is essential for this transition, as newly formed vessels supply oxygen and nutrient delivery while facilitating endochondral ossification and osteoprogenitor migration [1,3]. Disruption of vascular infiltration at this stage impairs mineralization and increases the risk of delayed union [1,3].

In the remodeling phase, woven bone is gradually replaced by lamellar bone through coordinated osteoclast and osteoblast activity. The vascular network undergoes parallel refinement to meet the metabolic demands of mature bone, highlighting the sustained importance of microvascular integrity throughout healing [1,3].

### 3.3. Angiogenesis and Microvascular Function in Skeletal Repair

Angiogenesis is a central determinant of successful fracture healing. Vascular disruption induces hypoxia, which activates pro-angiogenic pathways and drives new vessel formation within the developing callus. These vessels restore perfusion, enable oxygen and nutrient delivery, and provide structural and signaling support for bone formation [4,5]. Vascular invasion of the fibrocartilaginous callus is essential for endochondral ossification. VEGF produced by hypertrophic chondrocytes and osteogenic cells promotes endothelial sprouting and guides microvascular expansion, ensuring coordinated progression of angiogenesis and osteogenesis [5,21].

**Figure 2:**
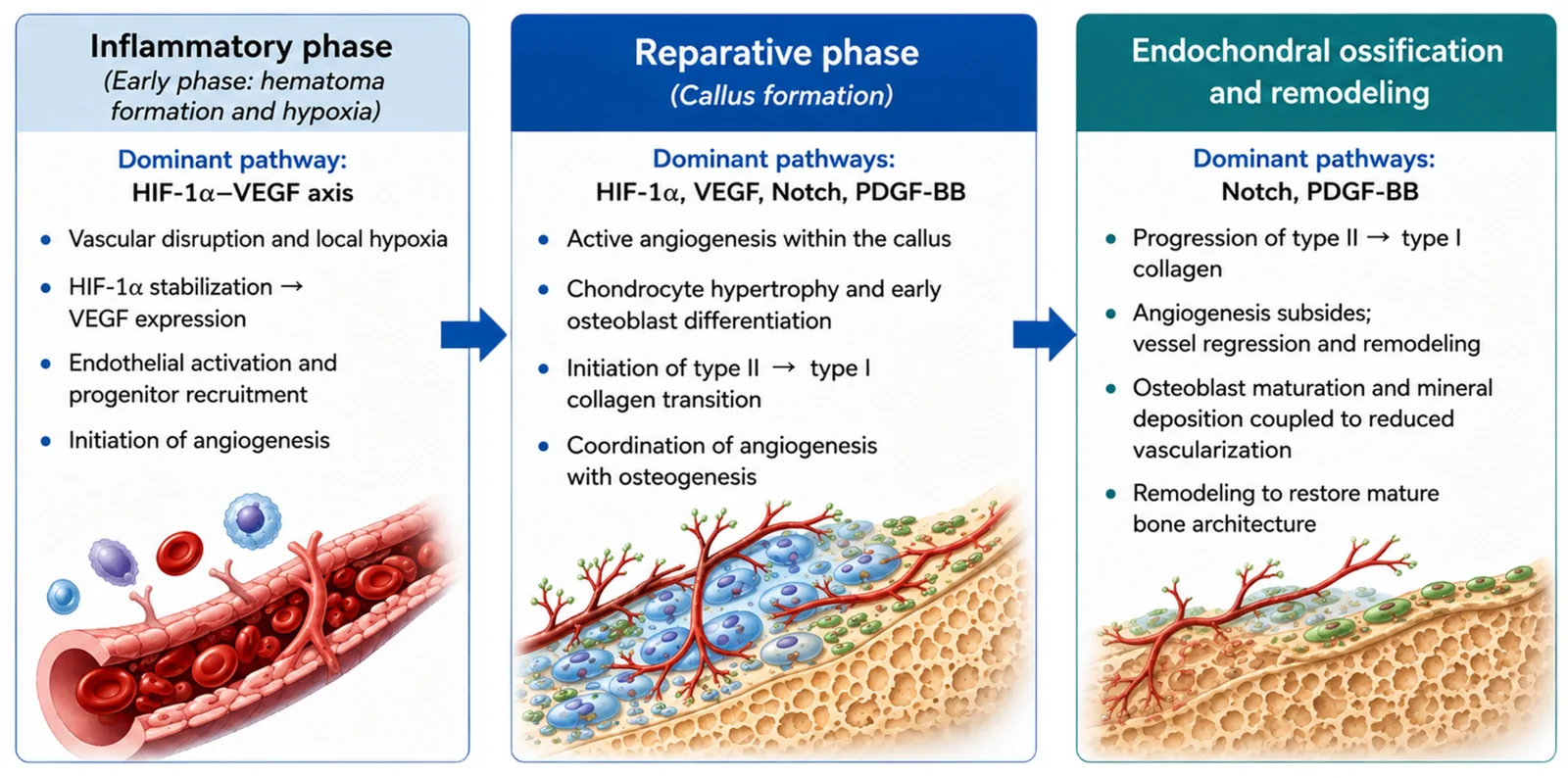
Phase-specific vascular and osteogenic signaling during fracture repair.

The extent and pattern of vascular development are further shaped by mechanical stability [28], oxygen tension [29], and inflammatory signaling [26,27]. Beyond perfusion, the microvasculature actively regulates skeletal regeneration through angiocrine signaling, supporting osteoblast differentiation and progenitor recruitment [20]. As healing progresses, the initially dense vascular network undergoes remodeling into an organized microcirculatory system adapted to mature bone (Figure 2).

### 3.4. Key Molecular Pathways

Among the most important regulatory systems are the hypoxia-inducible factor (HIF)–VEGF signaling axis, endothelial Notch signaling, and osteoclast-derived platelet-derived growth factor (PDGF)-BB, each active at a distinct but overlapping phase of repair, as summarized in Figure 2: HIF-1α–VEGF predominates during the initial inflammatory/hypoxic phase; Notch and PDGF-BB signaling become active during callus formation; and Notch/PDGF-BB signaling persists into endochondral ossification and remodeling as angiogenesis subsides.

One of the central mechanisms linking vascular growth and bone formation is the HIF–VEGF pathway. Following fracture, vascular disruption produces local hypoxia within the injured bone. Stabilization of HIF-1α under these conditions stimulates VEGF expression in osteoblasts, hypertrophic chondrocytes, and other bone-lineage cells, promoting endothelial cell migration, proliferation, and neovessel formation. This hypoxia-driven signaling ensures that vascular expansion occurs in parallel with the metabolic demands of newly forming bone tissue. Experimental studies demonstrate that stabilization of HIF-1α in osteoblasts, such as through deletion of the VHL gene, markedly increases VEGF production and enhances both angiogenesis and bone formation. Conversely, loss of osteoblast HIF-1α reduces vascular invasion and impairs skeletal regeneration, underscoring the importance of this pathway in coordinating oxygen sensing with vascular and osteogenic responses [30,31].

Endothelial Notch signaling represents another critical regulatory mechanism within the bone microvasculature, established primarily through studies of skeletal development and bone homeostasis in mice rather than adult fracture repair specifically. Unlike its generally inhibitory role in sprouting angiogenesis in many tissues, Notch signaling in bone endothelium promotes vascular proliferation and expansion of specialized microvascular populations [32,33]. In these developmental and homeostatic mouse models, experimental activation of endothelial Notch signaling increases vascular density and osteogenesis, whereas inhibition reduces bone angiogenesis and diminishes osteoprogenitor populations [32,34]. Whether this pathway operates comparably during adult fracture-callus angiogenesis has not been directly demonstrated.

One proposed mechanism underlying Notch’s role in these models involves the angiocrine secretion of Noggin by endothelial cells, positively regulated by Notch activity [32]. Noggin is a secreted BMP antagonist. Since BMP signaling normally drives osteoprogenitor differentiation, Noggin’s role is more precisely to transiently restrain premature differentiation, helping maintain an expandable pool of proliferating osteoprogenitors. Consistent with this, Noggin inhibits osteoblast differentiation from mesenchymal progenitors in vitro, and both loss and excess of endothelial/osteoblast-derived Noggin reduce bone mass in these mouse models, indicating that tightly regulated Noggin levels, not simple BMP antagonism alone, are required for normal bone formation [32]. Through this angiocrine signaling, endothelial cells help balance osteoprogenitor proliferation against differentiation, contributing to a vascular niche that supports bone regeneration in these model systems [27,30,34].

Bidirectional communication between endothelial and bone cells further supports this coupling. Osteoblasts promote vascular growth through angiogenic signaling, while endothelial cells release factors such as TGF-β and PDGF that support osteogenesis. In parallel, pre-osteoclast-derived PDGF-BB links bone resorption with angiogenesis by stimulating endothelial proliferation and progenitor recruitment [35].

Collectively, these signaling pathways illustrate a tightly regulated communication network between endothelial cells and bone cells. Hypoxia-driven HIF–VEGF signaling stimulates angiogenesis in response to metabolic demand, endothelial Notch signaling shapes vascular niches that support osteogenesis, and osteoclast-derived PDGF-BB links bone resorption with new vessel formation. Through these interconnected mechanisms, vascular and skeletal cells establish a coordinated signaling system that ensures adequate perfusion, nutrient delivery, and cellular recruitment during bone regeneration [34,35].

## 4. Linking Endothelial Dysfunction to Impaired Bone Repair

### 4.1. Impaired Angiogenic Signaling

This section examines whether dysregulated angiogenic signaling coordinated by the HIF-1α–VEGF axis and supported by PDGF-mediated remodeling links systemic ED to impaired fracture repair [30,35]. As detailed below, disruption of specific components of this cascade has been associated with reduced microvascular density and impaired endochondral ossification [36,37,38].

HIF-1α regulates post-fracture neovascularization by promoting VEGF expression in osteoblast-lineage cells under hypoxic conditions [30,34]. VEGF promotes endothelial sprouting and vascular invasion of the callus, coupling angiogenesis with mineralization [5]. Human studies have examined circulating VEGF as a biomarker during bone regeneration and after hip fracture, but available evidence remains observational and does not establish a causal relationship with fracture union [36,37], whereas reduced PDGF-AB levels have been observed in patients who later develop nonunion [38].

PDGF signaling contributes to endothelial proliferation and vascular stabilization, while pre-osteoclast-derived PDGF-BB links bone resorption with angiogenesis. Disruption of these processes may impair the coordination between cartilage resorption, vascular invasion, and osteoblast recruitment required for effective repair [5].

Endothelial Notch signaling contributes to vascular growth and osteogenic signaling in experimental models of bone development and homeostasis. Activation promotes type H vessel expansion and osteogenesis, whereas inhibition reduces both vascular density and osteoprogenitor populations [33]. This evidence derives from mouse models of skeletal development and bone homeostasis; whether comparable Notch-mediated vascular specialization occurs in the human fracture callus, and whether ED impairs it, remains speculative and has not been directly studied in fracture patients [32,34]. Endothelial progenitor cell (EPC) mobilization also contributes to post-fracture vascular regeneration; as this term encompasses several distinct cell populations (specified in Section 4.2), we identify the relevant population wherever possible. Circulating EPC levels increase after fracture but decline with age, and early-phase, non-randomized clinical trials using therapeutic CD34+ cell transplantation have reported faster union in nonunion cases compared with historical controls, though this remains preliminary rather than established evidence [39,40].

### 4.2. Endothelial Progenitor Cells

EPCs are considered a circulating reserve involved in vascular repair following injury. In patients with fractures, EPC levels increase rapidly within the first days after trauma and gradually decline over the following weeks, mirroring the early inflammatory and angiogenic phases of healing. This response appears to be attenuated in older individuals, suggesting an age-related reduction in regenerative capacity [41].

In adults, vasculogenesis remains an active process, with bone marrow derived progenitor cells contributing to new vessel formation by migrating to and integrating within sites of neovascularization [42]. Robust EPC mobilization has been demonstrated following acute ischemic and traumatic events: circulating CD34+ cell counts rise significantly after myocardial infarction, peaking around day 7 [43], and increase 6- to 12-fold after severe polytrauma [44]. Notably, this acute response contrasts with the impaired EPC numbers and mobilization capacity reported in chronic ischemic states such as critical limb ischemia [45], a distinction that parallels the age- and disease-related EPC impairments discussed below. ED adversely affects both the number and functional capacity of these progenitor cells.

The term EPC has been applied inconsistently to several distinct cell populations; we specify the relevant population wherever the underlying study allows. Clinical studies most commonly use either broadly defined circulating CD34+ cells a heterogeneous hematopoietic population, only a minority of which have endothelial potential or the narrower CD34+/KDR+ phenotype intended to enrich for endothelial specificity [46]. Both are considered part of a circulating reserve involved in vascular repair following injury.

Lower EPC levels and impaired angiogenic potential have been consistently observed in individuals with cardiovascular risk factors, including diabetes and smoking [46,47,48], though these studies were conducted in general cardiovascular populations rather than fracture patients. Whether comparable EPC impairment occurs at the fracture site in these same individuals has not been directly tested; the link to healing outcomes remains inferential.

At the same time, the concept of EPCs is not without limitations. There is ongoing debate regarding their precise definition and measurement, and no consensus marker set currently exists to unambiguously identify a “true” endothelial progenitor [49]. Frequently used methods vary substantially in what they actually capture. Broadening the phenotype from CD34+/KDR+ to CD34+/CD133+/KDR+ was intended to enrich for a more primitive, endothelial-specific population, but this triple-positive fraction is now recognized to overlap heavily with hematopoietic progenitors and fails to reliably form vessels in vivo [50]. Colony-forming unit (CFU-Hill) assays capture a functionally distinct population still further: these cells express some endothelial-associated markers but are predominantly myeloid/monocytic in origin and lineage [50]. By contrast, endothelial colony-forming cells (ECFCs), identified through late-outgrowth culture assays, are clonogenic, committed to endothelial lineage, and capable of forming functional vessels in vivo, making them the population most consistent with the original EPC concept, though rarely used in the clinical fracture-healing literature reviewed here [50]. In addition, functional properties differ substantially across assay types, limiting direct comparison between studies [49]. For this reason, EPC-related measurements are more appropriately interpreted as indirect indicators of vascular repair capacity rather than as a definitive assessment of endothelial progenitor cells.

Clinical observations further illustrate, but do not establish, the potential relevance of progenitor-mediated vascular repair. Transplantation of autologous CD34+ cells, the broadly defined hematopoietic population, not a narrower endothelial-specific phenotype, has been evaluated in an initial pilot trial in seven patients [40] and a subsequent trial in 25 patients [39]; both reported faster union compared with historical controls. Neither trial included a concurrent randomized control group, and both were conducted by the same research team; the larger trial’s phase III designation should not be read as equivalent to a large randomized controlled trial. These findings are best characterized as encouraging but preliminary, not as established evidence that augmenting progenitor-driven vascular responses improves fracture repair. Substantial heterogeneity in EPC characterization, study design, and patient selection further limits generalizability, and independent, randomized, controlled trials are needed before therapeutic conclusions can be drawn [40,46,51].

### 4.3. Oxidative Stress and Progenitor Cell Dysfunction

Oxidative stress is considered a key driver of ED and may impair vascular competence during fracture repair. Excess reactive oxygen species reduce nitric oxide bioavailability and promote endothelial activation, contributing to impaired angiogenesis [16,17].

A central mechanism is endothelial nitric oxide synthase (eNOS) uncoupling, in which oxidative depletion of tetrahydrobiopterin shifts nitric oxide (NO) synthase activity toward superoxide production, amplifying oxidative stress and reducing NO signaling [52]. Elevated levels of ADMA further inhibit NO production and are associated with impaired endothelial function in clinical populations [53]. These mechanisms are established in general vascular and cardiovascular populations; direct evidence of eNOS uncoupling or ADMA elevation within fracture callus tissue itself is currently lacking.

Oxidative stress also directly affects EPCs by promoting senescence and reducing proliferative and migratory capacity. Mitochondrial dysfunction further exacerbates this process by impairing cellular metabolism and survival. Together, these mechanisms limit effective neovascularization during early callus formation [51,54].

Systemic oxidative stress has also been linked to reduced bone mineral density and altered remodeling, suggesting combined vascular and skeletal effects that may further impair fracture repair [55].

### 4.4. Nitric Oxide Deficiency and Osteogenesis

Nitric oxide plays a role in both endothelial function and osteogenesis in preclinical models, though this mechanistic promise has not translated into demonstrated clinical benefit, as detailed below. Experimental mouse models demonstrate that reduced nitric oxide availability impairs osteoblast differentiation and mineralization, partly through disruption of metabolic pathways required for anabolic activity [56].

Clinical evidence for NO-based therapy in bone health is weak. A methodologically sound randomized trial found no prevention of early postmenopausal bone loss [57]. No adequately powered human trial currently demonstrates a clinical benefit of NO-based therapy for bone density or fracture prevention. Despite biologic plausibility from preclinical models, current evidence does not support any role for nitric oxide-based therapy in human fracture prevention or bone healing.

Genetic studies similarly indicate that minor variation in nitric oxide signaling has limited impact on skeletal outcomes [58]. In contrast, acquired ED driven by oxidative stress, inflammation, and metabolic disease is biologically plausible as a determinant of local nitric oxide deficiency in fracture healing, though this pathway has not been directly demonstrated in fracture patients.

### 4.5. Inflammatory Endothelial Phenotype

An inflammatory endothelial phenotype represents an important interface between systemic vascular dysfunction and impaired fracture healing. Following fracture, a controlled inflammatory response is required to initiate tissue repair. In uncomplicated healing, inflammatory markers such as C-reactive protein (CRP) and interleukin-6 (IL-6) rise acutely after injury and subsequently decline as the reparative phase progresses. Large clinical fracture cohorts demonstrate that CRP follows a predictable postoperative trajectory, peaking early and gradually decreasing in the absence of complications [59]. This transient inflammation reflects a physiological component of the healing response rather than pathological inflammation.

In contrast, persistent systemic inflammation appears to be associated with impaired healing trajectories. In a clinical study of patients undergoing tibial fracture surgery, individuals who developed delayed healing exhibited significantly higher circulating levels of IL-6, CRP, and tumor necrosis factor-α (TNF-α) compared with those achieving normal union [60]. Multivariable analyses further demonstrated that elevated inflammatory markers independently correlated with an increased risk of delayed healing. These findings provide direct human evidence linking sustained systemic inflammation with impaired fracture repair.

The relevance of this inflammatory state extends beyond overt infection. In a prospective cohort study of patients undergoing revision surgery for presumed aseptic long-bone nonunion, elevated CRP levels were frequently observed despite the absence of clinical signs of infection [61]. Although some patients demonstrated microbiological positivity on intraoperative sampling, CRP alone showed limited predictive accuracy for occult infection. Importantly, elevated inflammatory markers were not consistently attributable to identifiable pathogens. These findings suggest that impaired fracture repair may be associated with persistent systemic inflammation even in cases classified as aseptic. Rather than reflecting purely infectious complications, chronic low-grade inflammation may represent an intrinsic component of impaired reparative biology.

Hip fracture cohorts further support the clinical importance of systemic inflammatory signaling. In elderly patients undergoing hip fracture surgery, circulating IL-6 and CRP concentrations rise markedly in the early post-fracture period, and higher admission cytokine levels are associated with poorer clinical recovery trajectories [62,63]. Although these studies primarily evaluate postoperative outcomes rather than radiographic union, they demonstrate that exaggerated inflammatory responses following fracture are clinically meaningful and may reflect broader disturbances in systemic physiology.

From a biological perspective, sustained inflammatory signaling may disrupt the transition from the inflammatory phase of fracture repair to the angiogenic reparative phase. Fracture healing is a temporally regulated process in which early inflammatory activation must resolve to permit angiogenesis and callus maturation [1]. Persistent cytokine signaling has been associated with altered expression profiles in cases of delayed union and nonunion, suggesting that failure to appropriately resolve inflammation may impair vascular invasion and destabilize endochondral ossification [26].

Temporal dysregulation of inflammatory signaling appears particularly important, as altered cytokine kinetics have been observed in patients with impaired healing [64].

From a mechanistic standpoint, persistent inflammation is known to promote endothelial activation and impair microvascular stability in other tissue contexts; whether this endothelial pathway mediates the CRP/IL-6–delayed healing association described above, as opposed to inflammation acting through other reparative pathways, has not been directly assessed.

## 5. Clinical Conditions Supporting the Vascular–Bone Link

The systemic conditions discussed below are known to impair fracture healing through multiple parallel pathways, only some of which are mediated by endothelial dysfunction. Where established, we note non-vascular contributing mechanisms; direct effects on osteoblasts and chondrocytes, bone matrix quality, mineral metabolism, neuropathy, medication effects, and prior treatment history alongside the vascular pathways that are this review’s primary focus. These conditions, their principal endothelial mechanisms, and the type and quality of supporting evidence are summarized in Table 1.

### 5.1. Diabetes Mellitus

Diabetes mellitus is associated both with increased fracture risk (fragility fracture incidence) and, distinctly, with impaired fracture repair. This section focuses on the repair-related evidence.

Clinical studies show higher rates of delayed union and nonunion, particularly with long disease duration or poor glycemic control. This cannot be explained by reduced bone mineral density alone, as diabetes also alters bone quality and disrupts reparative pathways.

Chronic hyperglycemia promotes accumulation of advanced glycation end products within the bone collagen matrix, impairing cross-linking, reducing viscoelasticity, and increasing fragility. Diabetic peripheral neuropathy independently contributes to impaired healing through reduced protective sensation, altered mechanical loading, and disrupted autonomic regulation of bone blood flow and remodeling a pathway distinct from, though potentially compounding, endothelial dysfunction [74]. Diabetes-related skeletal pathology also involves disrupted cellular activity and vascular repair [10]. ED represents one of several contributing mechanisms in this process. Through oxidative stress, inflammation, and reduced NO bioavailability, diabetes impairs vasodilation and promotes microvascular dysfunction in the systemic vasculature generally [23]. Whether this extends to comparable microvascular impairment within bone or the fracture callus specifically has not been directly measured; the effect on local perfusion is inferred rather than demonstrated.

Diabetes also prolongs and dysregulates the inflammatory phase of healing. Impaired macrophage transition from pro-inflammatory to reparative phenotypes reduces release of mediators such as VEGF, weakening angiogenesis and delaying progression to the reparative phase [10]. As a result, vascular invasion of the callus, endochondral ossification, and remodeling are compromised. Diabetes additionally impairs osteoblast function, alters osteoclast activity [74], and disrupts repair responses, with delayed or reduced increases in osteocalcin, alkaline phosphatase, and IGF-1 [75]. Collectively, these findings are consistent with a model in which metabolic dysregulation, ED, and impaired angiogenic signaling compromise skeletal repair in diabetes.

### 5.2. Aging and Vascular Aging

Aging is associated with impaired fracture healing, plausibly through disrupted coordination between angiogenesis and bone regeneration, though the underlying VEGF/HIF-1α biology is more nuanced than a uniform age-related decline. Notably, a prospective human study measuring VEGF and PDGF directly in fracture hematoma found that angiogenic cytokine levels and biological activity were preserved in elderly patients compared with younger patients, suggesting that growth factor deficiency is not the sole explanation for impaired vascularization in elderly nonunion, and that impaired cellular responsiveness to otherwise-adequate angiogenic signals may better account for it [65]. Increased oxidative stress, elevated NADPH oxidase activity, reduced nitric oxide availability, and mitochondrial dysfunction further impair endothelial function with age; these are established features of vascular aging broadly, though direct confirmation within the aging fracture-healing microenvironment specifically is limited. Aging also reduces the responsiveness of skeletal stem and progenitor cells to angiogenic and osteogenic stimuli, consistent with this cellular-responsiveness explanation, so dysfunction in both compartments may act together to delay repair [66,76].

Structural and functional vascular changes further limit healing in older adults. Reduced skeletal blood flow, decreased microvascular density, and impaired vasodilatory responses restrict delivery of oxygen, nutrients, and progenitor cells to the fracture site. These deficits provide a basis for weakened angiogenic responses, delay callus vascularization, and impair endochondral ossification. Clinically, this manifests as delayed union, prolonged immobilization, and higher complication rates [76]. Overall, vascular aging blunts neovascularization and reduces microvessel maturation, making angiogenesis a major limiting factor in fracture healing in older adults [77].

Independent of vascular and progenitor-cell aging, sarcopenia, reduced mechanical loading, declining sex hormone levels, and impaired nutritional status also contribute substantially to age-related healing impairment [76].

### 5.3. Chronic Kidney Disease

In chronic kidney disease (CKD), ED arises from inflammatory, oxidative, and hemodynamic disturbances that compromise vascular integrity and repair. Uremic toxins directly injure endothelial cells and impair vascular homeostasis [68], based on general vascular biology rather than bone-specific data. Because bone depends on intact vascular supply, CKD-related endothelial injury plausibly extends to bone perfusion, a link directly supported, in this case, by experimental bone perfusion data below [67]. Reduced cardiac output and vascular calcification further compromise bone perfusion. Experimental high-turnover CKD models show site- and stage-dependent alterations in bone perfusion, with early increases in cortical perfusion followed by declines in marrow perfusion in advanced disease. These later changes are associated with CKD–mineral and bone disorder (CKD-MBD), including elevated PTH, secondary hyperparathyroidism, marrow fibrosis, and reduced VEGF signaling. Notably, marrow fibrosis is a well-established consequence of CKD-MBD that operates largely independently of vascular perfusion changes, alongside reduced VEGF signaling [67].

### 5.4. Smoking as a Modifiable Risk Factor

Clinical and experimental studies show delayed fracture healing in smokers. Smoking is associated with reduced tissue blood flow, lower oxygen tension, and impaired angiogenic and osteogenic signaling. Nicotine contributes by reducing BMP-2 and VEGF expression and suppressing TGF-β, impairing mesenchymal cell recruitment, proliferation, and differentiation and slowing ossification.

Smoking also disrupts the vitamin D–parathyroid hormone axis. Vitamin D deficiency delays fracture healing and smoking further reduces calcium and vitamin D absorption. Parathyroid hormone promotes bone regeneration and stimulates angiogenesis, yet circulating PTH levels are generally lower in smokers [69].

Smoking also disrupts the early inflammatory phase of repair and interferes with chondrogenesis, osteoblast differentiation, and osteogenesis. Cigarette smoke alters the expression of genes such as alkaline phosphatase, BMP-2, receptor activator of nuclear factor κB ligand (RANKL), and osteoprotegerin. Fibroblast migration into fracture sites is impaired, limiting extracellular matrix deposition and growth factor release required for neovascularization. Clinically, smokers experience longer time to union, higher nonunion rates, increased need for bone grafting, and worse outcomes after long-bone fractures and spinal fusion. At the vascular level, nicotine and carbon monoxide reduce microperfusion, damage endothelial cells, increase blood viscosity, and promote hypercoagulability, leading to microvascular thrombosis. Smoking also reduces osteogenic and angiogenic mediators including TGF-β, VEGF, PDGF, and prostacyclin [70].

These vascular effects operate alongside smoking’s well-documented direct effects on osteoblast gene expression and mesenchymal cell recruitment described above, rather than as the sole mechanism.

### 5.5. Obesity

Obesity is characterized by excess adipose tissue that promotes chronic inflammation through increased secretion of IL-6, IL-1β, TNF-α, leptin, and MCP-1, along with reduced adiponectin. These disturbances contribute to ED through imbalance between vasodilators including NO, EDHF, and prostacyclin and vasoconstrictors such as endothelin-1, angiotensin II, and prostaglandin H_2_. Reduced NO bioavailability, endothelial NO synthase uncoupling, increased oxidative stress, and activation of inflammatory pathways including NF-κB, mitogen-activated protein kinases (MAPKs), and the NLR family pyrin domain containing 3 (NLRP3) inflammasome contribute to vascular inflammation and remodeling [71].

Obesity also alters bone metabolism. Bone marrow mesenchymal stem cells preferentially differentiate into adipocytes rather than osteoblasts, partly because increased sFRP1 suppresses Wnt signaling and reduces osteogenic commitment. Elevated lipids, inflammation, and oxidative stress further promote osteoclastogenesis through RANK–RANKL signaling. Clinically, obesity is associated with delayed fracture healing, higher nonunion rates, and reduced likelihood of union within 18 months. Mechanistically, delayed repair reflects impaired mesenchymal stem cell function, reduced FGFR2 expression, excessive TNF-α production, and increased osteoclast activity [78].

These mechanisms are described in general metabolic and cardiovascular contexts [71]; their specific contribution to bone microvascular impairment in obese fracture patients, as distinct from obesity’s direct osteogenic and mechanical effects, has not been isolated.

### 5.6. Chronic Inflammatory Diseases

Patients with HIV infection experience higher rates of fragility fractures, a fracture-risk outcome, alongside reports of delayed bone repair. Autoimmune diseases such as systemic lupus erythematosus and rheumatoid arthritis are separately associated with impaired fracture healing specifically [79].

In rheumatoid arthritis, systemic inflammation contributes directly to skeletal fragility. Bone mineral density declines with disease severity, and glucocorticoid therapy further increases fracture risk. Pro-inflammatory cytokines such as TNF-α and IL-6 activate RANKL signaling, promoting osteoclastogenesis while inhibiting osteoblast differentiation. Reduced bone mineral density in RA has also been directly associated with impaired systemic endothelial function (reactive hyperaemia index, pulse wave velocity) in a cross-sectional cohort [72]; note that this evidence concerns bone density, not fracture healing, and a comparable direct measurement in RA-related fracture repair is lacking although animal nonunion data below [73] address healing specifically. Endothelial nitric oxide signaling supports vascular integrity and bone turnover and is impaired in RA alongside estrogen deficiency and oxidative stress [80]. Separately, RA is associated with an elevated RANKL/OPG ratio reflecting increased bone resorption [81]. Reduced blood flow to bone may limit nutrient delivery and impair calcification and repair.

Animal models support this relationship. In serum-transfer-induced rheumatoid arthritis models, sustained inflammation is associated with fracture nonunion, reduced callus formation, fibrotic tissue deposition, and impaired angiogenesis, hindering fracture repair [73].

### 5.7. Medication-Related Confounding

Glucocorticoid exposure exemplifies medication-induced disruption of vascular–bone coupling. In experimental models, chronic prednisolone reduces trabecular bone volume, femur length, and growth plate height, with defects appearing within two weeks. Glucocorticoids also reduce type H vessels, impair vessel branching and maturation, disrupt chondrocyte differentiation, increase apoptosis, suppress growth hormone (GH)/insulin-like growth factor 1 (IGF-1) signaling, and reduce vascular invasion into the growth plate, findings based on murine growth-plate models rather than adult fracture-callus tissue [82]. Because type H vessels are closely associated with osteoprogenitor recruitment and bone formation, their decline may directly impair osteogenesis.

Glucocorticoids also suppress PDGF-BB, a key factor supporting endothelial progenitor migration and type H vessel formation. Through interference with NF-κB-dependent regulation of the Pdgfb promoter, they impair skeletal angiogenesis and remodeling [83].

In addition, glucocorticoids reduce circulating EPCs and impair their migration, angiogenic activity, homing, and resistance to senescence. In steroid-induced osteonecrosis of the femoral head, these changes are accompanied by endothelial injury, coagulation–fibrinolysis imbalance, thrombus formation, reduced VEGF expression, osteocyte apoptosis, and decreased skeletal vessel volume [82,83]. A rat model of glucocorticoid-induced femoral head osteonecrosis found that Vitamin K2 partially protected against this vascular damage, offering early preclinical evidence for a possible protective strategy, though this has not been tested clinically [84]. Collectively, these findings suggest that glucocorticoids disrupt vascular and skeletal regeneration, reinforcing the interdependence of angiogenesis and osteogenesis during bone repair. It is also worth noting that glucocorticoid-related skeletal vulnerability may reflect cumulative lifetime exposure rather than current use alone, for example, corticosteroid or chemotherapy exposure during childhood cancer treatment can affect adult bone status independently of any present-day vascular findings.

The principal endothelial and angiogenic pathways discussed above, together with their roles in fracture repair and the main limitations of the supporting evidence, are summarized in Table 2.

## 6. Cardiovascular Disease and Orthopedic Outcomes

### 6.1. Fracture Healing Outcomes in Patients with Cardiovascular Disease

This section examines a specific and mechanistically well-characterized example of cardiovascular pathology affecting a type H vascular niche: acute myocardial infarction. This acute ischemic model differs importantly from the chronic, low-grade endothelial dysfunction associated with diabetes, aging, CKD, smoking, and obesity discussed in Section 5, and readers should be cautious about extrapolating these findings to those more typical fracture-patient populations.

Myocardial infarction induces alterations in the bone marrow vascular niche in both mice and humans, with a selective impact on type H endothelial cells responsible for coupling angiogenesis and osteogenesis. In a mouse model using left anterior descending coronary artery ligation, type H endothelial cells declined over time and reached significance by 28 days after infarction, while type L endothelial cells showed no change. This reduction was independent of age and coincided with expansion of long-term hematopoietic stem cells and myeloid-biased progenitors with increased CD41 expression. In human bone marrow aspirates from post-infarction heart failure patients, analyses showed a selective decrease in CD31^hi^EMCN^hi^ endothelial cells while total endothelial cell numbers remained stable. Single-cell RNA sequencing demonstrated elevated expression of inflammatory genes, including IL1B and MYC, within type H endothelial cells, alongside preserved endothelial marker expression, supporting an inflammation-driven phenotypic shift [85].

Mechanistic experiments indicated that vascular niche deterioration after myocardial infarction is driven primarily by inflammatory signaling. Pharmacologic blockade of β2-adrenergic receptors did not prevent type H endothelial cell loss or myeloid progenitor expansion. In contrast, inhibition of IL-1β production through blockade of the NLRP3 inflammasome partially preserved type H vasculature and reduced myeloid progenitor expansion. These findings suggest IL-1β-dependent activation of MYC in type H endothelial cells promotes endothelial cell death and depletion of this osteogenic subtype, linking myocardial infarction-induced inflammation with impaired bone marrow endothelial function and altered hematopoiesis [85].

Two caveats limit direct extrapolation of these findings to fracture healing more broadly. First, this model describes an acute, IL-1β/NLRP3-driven inflammatory insult unfolding over days to weeks after a discrete ischemic event, a temporal and mechanistic profile distinct from the chronic ED states discussed elsewhere in this review. Second, the vascular niche examined is the bone marrow hematopoietic niche, not the fracture callus itself; whether comparable type H endothelial cell depletion occurs at an actual fracture site, or in patients with chronic ED but no recent acute cardiovascular event, has not been directly studied [85]. These findings are best read as proof-of-principle that cardiovascular pathology can disrupt type H vasculature via inflammatory mechanisms, not as direct evidence that chronic ED impairs fracture healing through this specific pathway.

More broadly, myocardial infarction represents an acute ischemic injury followed by a systemic inflammatory response. Cardiovascular and skeletal systems share overlapping regulatory pathways, including RANKL–OPG signaling, BMP, and mineral-regulating proteins. After infarction, inflammatory cytokines such as IL-1, TNF-α, and IL-6 increase systemically and stimulate osteoclast activity. Experimental models show that both myocardial infarction and fracture trigger cytokine cascades that reduce trabecular bone volume at distant skeletal sites. Additional mechanisms involve neuroimmune and complement-mediated pathways. Activation of the sympathetic nervous system after infarction increases monocyte production and recruitment, contributing to vascular pathology and bone resorption. Complement signaling through C5a–C5aR also promotes osteoclastogenesis and inflammatory amplification. Taken together, these findings support myocardial infarction as a systemic inflammatory event that alters bone remodeling and vascular–skeletal homeostasis [86].

Among cardiovascular conditions, mechanisms most plausibly linked to impaired fracture repair include systemic inflammation, reduced tissue perfusion, ED, and frailty-associated reductions in physiologic reserve. Other cardiovascular comorbidities, including atrial fibrillation, may function primarily as markers of overall cardiovascular vulnerability rather than direct determinants of impaired bone healing.

### 6.2. Post-Fracture Morbidity and Mortality

Cardiovascular comorbidity also shapes broader recovery after fracture, though largely through outcomes distinct from bone union itself. In elderly hip fracture patients, heart failure independently predicts poorer functional recovery [87], and atrial fibrillation is associated with increased perioperative complications and mortality [88]. More broadly, cardiovascular disease is linked to greater postoperative morbidity after orthopedic trauma [89]. These associations reflect shared vulnerability; inflammation, impaired perfusion, and reduced physiologic reserve rather than a demonstrated direct effect on fracture-site healing.

### 6.3. Perioperative Cardiovascular Risk After Fractures

Perioperative myocardial injury and atrial fibrillation are common after hip fracture surgery, frequently clinically silent, and associated with increased mortality [90,91,92]. Beyond their prognostic significance, these events signal systemic hypoxia, hemodynamic instability, and impaired perfusion that could plausibly affect endothelial integrity and microvascular regulation during early healing, though this link has not been directly tested in fracture-healing outcomes.

### 6.4. Hip Fracture as a Cardiovascular Stressor

Long-bone fracture triggers a systemic inflammatory and complement-mediated response including C5a, IL-6, TNF-α, and extracellular histones that extends to biochemical evidence of myocardial stress in both animal and human studies [93]. C5a–C5aR signaling promotes oxidative stress, cytokine release, and endothelial activation [93]. This systemic activation may plausibly disrupt endothelial function during early fracture healing, particularly in elderly or cardiopulmonary-compromised patients [94], though direct evidence linking this cascade to callus vascularization is currently lacking.

### 6.5. Evidence Predominantly Associative Rather than Causal

The following studies address fracture risk and incidence rather than post-fracture repair, and are included as supporting epidemiological context.

In this cross-sectional analysis of 17,606 adults, higher cardiovascular health scores were associated with lower prevalence of osteoporotic fractures. Each one-point increase in the LE8 score corresponded to a 1% reduction in fracture odds. Individuals with moderate and high cardiovascular health had 22% and 34% lower odds of fractures compared with those with low scores [95]. This relationship remained significant after adjustment for demographic and health-related variables and was consistent across subgroups.

Several cardiovascular health components showed inverse associations with fracture prevalence, including physical activity, diet quality, tobacco exposure, body mass index, and blood pressure. In contrast, blood glucose, lipid levels, and sleep showed weaker or inconsistent associations. Overall, these findings provide associative evidence linking better cardiovascular health with lower skeletal fragility without implying causation.

In an analysis of 47,437 Medicare patients with 56,492 fractures, 2.5% progressed to nonunion. Nonunion was more frequent in younger patients, reaching 5.5% in those aged 55–59, compared with 1.3% in patients aged 85 and older. Rates varied by fracture site, peaking at 6.4% in scaphoid fractures and remaining low in ribs and trunk. Smoking, obesity, rheumatoid arthritis, diabetes, alcoholism, osteoporosis, and open fractures were associated with nonunion, while age over 75 showed an inverse association [96]. A history of cardiovascular disease or stroke was linked to lower nonunion risk, and overall comorbidity burden did not independently predict nonunion.

The same Medicare cohort also reported mortality outcomes, which reflect post-fracture survival rather than healing. Fracture was associated with increased one-year mortality across multiple skeletal sites, with femur and pelvic fractures showing more than threefold higher mortality compared with individuals without fracture. Predictors of death included stroke, vascular disease, morbid obesity, phlebitis, alcoholism, malnutrition, and smoking. The inverse association between nonunion and early mortality suggests competing risks, as some patients died before nonunion diagnosis. These results highlight interactions between age, comorbidity, and bone healing outcomes.

In a cohort of 414 individuals with high rates of hypertension, dyslipidemia, and diabetes, estimated fracture risk assessed by FRAX was associated with vascular dysfunction and arterial changes. Higher FRAX scores correlated with reduced flow-mediated dilation, reduced nitroglycerin-induced dilation, and increased brachial artery intima-media thickness. These associations persisted after adjustment for cardiovascular risk factors, indicating an independent relationship between fracture risk and impaired vascular function in both sexes.

The associations were stronger in women, and fracture risk remained linked to vascular dysfunction after adjustment for global cardiovascular risk scores. Increasing FRAX scores were associated with worsening endothelium-dependent and -independent vasodilation. These findings provide evidence that higher fracture risk parallels ED and arterial remodeling, providing a possible relationship between skeletal fragility/fracture risk and vascular health [97], this reflects fracture risk rather than post-fracture repair.

### 6.6. Potential Bidirectional Relationship

A separate but related literature links cardiovascular disease to fracture risk and bone mineral density, distinct from fracture healing. Large epidemiological cohorts and Mendelian randomization studies show bidirectional associations between low BMD/fracture and cardiovascular disease [98,99], with heart failure and vascular pathology also linked to altered bone turnover markers [100]. These findings support shared pathophysiology (inflammation, oxidative stress, ED) but speak to skeletal fragility and fracture incidence rather than post-fracture repair, and are included here only as supporting context.

## 7. Importance of Cardiovascular Health in Fracture Patients

Bone repair occurs within a broader systemic environment rather than as a purely localized process: long-bone fractures trigger inflammatory, neuroendocrine, and cardiovascular responses that extend beyond the injured tissue [93], particularly relevant in older adults with preexisting cardiovascular disease, ref. [89,91] the perioperative complications discussed in Section 6.2 and Section 6.3. Their implications may extend beyond perioperative mortality: impaired systemic perfusion and inflammatory/endothelial activation could plausibly interfere with the angiogenic processes central to fracture healing (Section 3), though this specific link remains untested. This parallels the fracture-risk, rather than fracture-repair, evidence already discussed in Section 5.6 and Section 6.5 [72,99] reinforcing that cardiovascular health is best understood as part of the fracture patient’s systemic environment, not an isolated comorbidity.

### 7.1. What This Review Does Not Recommend

Although ED is biologically relevant and measurable using several clinical and laboratory methods (Table 3), current evidence does not support routine endothelial testing in fracture patients, and no professional guideline currently recommends it. No validated predictive threshold links any measure of endothelial function to fracture-healing outcome, and no prospective trial has shown that identifying ED in a fracture patient changes management or improves outcomes [13,15]. Biomarker-guided endothelial testing should therefore be confined to research protocols, not offered as clinical care, until such evidence exists.

Similarly, angiogenic and vascular-targeted therapies remain experimental. Despite the promising preclinical data described throughout this review, no angiogenic therapy has been shown in an adequately powered human trial to improve fracture healing, and off-label use for this purpose is not supported by current evidence and should not be undertaken outside a clinical trial. Cardiovascular medications should continue to be prescribed strictly according to their established clinical indications, not for speculative orthopedic benefit.

### 7.2. Relevance for Multidisciplinary Orthopedic–Cardiology Care

The emerging evidence linking vascular dysfunction and fracture healing also has practical implications for clinical care. Because older fracture patients frequently experience perioperative cardiovascular instability [88,90,91], closer collaboration between orthopedic and cardiovascular specialists may be valuable in medically complex cases. Non-invasive vascular assessments such as flow-mediated dilation are already standardized in cardiovascular medicine [24] and could be explored in high-risk fracture research cohorts, though no validated clinical pathway currently exists for their use in fracture care.

Importantly, recognizing this shared pathophysiology does not imply that endothelial testing should become routine in fracture care. Rather, it highlights the value of multidisciplinary perspectives when evaluating patients whose recovery may be influenced by both skeletal injury and systemic vascular disease.

## 8. Limitations of Current Evidence and Future Research Needs

Despite increasing recognition of the link between ED and impaired bone repair, important limitations still constrain causal interpretation. Much of the current evidence remains indirect and largely derived from preclinical models. Experimental and cellular studies have been essential in defining how angiogenic pathways such as VEGF-driven vascular invasion, HIF-1α-mediated hypoxic responses, and endothelial signaling networks coordinating angiogenesis with osteogenesis support fracture healing [21]. These models consistently show that impaired angiogenesis disrupts callus formation and compromises regeneration. However, their relevance to clinical practice is limited. Most are performed in young, otherwise healthy animals under tightly controlled conditions that do not reflect the complexity of typical fracture patients, where aging, cardiometabolic disease, smoking, and systemic vascular dysfunction frequently coexist. As a result, direct human data examining ED as a determinant of fracture healing remain limited, leaving a clear translational gap.

A further challenge lies in the heterogeneity of study design across disciplines. Research on ED is usually conducted within cardiovascular frameworks, using measures such as flow-mediated dilation, circulating biomarkers, or EPC counts, whereas orthopedic studies focus on structural endpoints, including callus formation, time to union, and mechanical stability. Because these approaches rarely overlap, direct comparisons are difficult. In practice, most orthopedic studies do not include direct assessments of endothelial or microvascular function at the fracture site, meaning vascular contributions are often inferred rather than directly demonstrated.

Interpretation is also complicated by the timing of measurements. Many clinical studies assess vascular biomarkers after the fracture has occurred, when systemic inflammation, oxidative stress, and transient endothelial activation are already present. Under these conditions, altered biomarker levels may reflect the physiological response to injury rather than a preexisting abnormality. This makes it difficult to establish directionality and highlights the need for prospective studies capturing vascular status before, or very early after, injury.

There is also the possibility of publication bias shaping the field. Experimental work tends to emphasize pathways that enhance angiogenesis and bone formation, while neutral or negative findings are less frequently reported. This can skew the literature toward particular pro-angiogenic mechanisms, especially in controlled preclinical settings, and may exaggerate their relative importance.

Taken together, these limitations the preclinical-to-clinical translational gap, cross-disciplinary measurement heterogeneity, timing-related confounding, and potential publication bias define the specific methodological barriers future research must overcome; we outline the resulting study-design priorities in the Conclusions and Future Perspectives section below.

## 9. Conclusions and Future Perspectives

Fracture healing depends not only on osteogenic activity but also on coordinated restoration of the vascular microenvironment. Endothelial cells contribute to skeletal repair through angiogenesis, oxygen and nutrient delivery, and angiocrine signaling supporting osteoprogenitor recruitment [4,6]. Disruption of these processes can impair angiogenic–osteogenic coupling, and ED represents a plausible, though unproven, systemic contributor to delayed union or nonunion.

Several mechanisms support this including relationship with reduced NO bioavailability, oxidative stress, inflammatory endothelial activation, and impaired EPC mobilization acting through HIF-1α–VEGF signaling, PDGF-mediated remodeling, and endothelial Notch signaling (as detailed in Section 3.4). Clinically, diabetes, aging, CKD, obesity, smoking, and chronic inflammatory disease are each associated with impaired healing, though as emphasized throughout Section 4, Section 5 and Section 6 largely through associative rather than direct mechanistic evidence, and via multiple parallel pathways of which ED is only one (Section 5 introduction).

Advances in endothelial biology add further nuance: substantial cellular heterogeneity exists across endothelial populations [12,19], and bone-marrow-specific vascular niches regulating osteoprogenitors may help explain variable healing outcomes among similar injuries, a heterogeneity compounded by aging-related declines in both vascular and progenitor-cell responsiveness (Section 5.2).

Clarifying ED’s causal contribution will require prospective cohorts that characterize vascular status via circulating biomarkers or functional testing such as flow-mediated dilation [24] at or near fracture presentation, paired with standardized healing endpoints (radiographic union, time to weight-bearing, defined nonunion criteria), as outlined in Section 8. Endothelial biomarkers, largely unstudied in fracture cohorts to date, could complement rather than replace established clinical risk factors in stratifying healing risk.

Ultimately, progress will depend on integrated research frameworks spanning vascular biology, endocrinology, and orthopedic trauma science. Recognizing ED as one plausible contributor, not the primary explanation to impaired bone regeneration, may help refine risk stratification and identify novel therapeutic targets, provided this promise is tested against the cautions raised throughout this review (Section 7.1).

Key terms and abbreviations used throughout this review are defined in Table 4.

## Figures and Tables

**Table 1:** Systemic conditions linking ED to impaired fracture healing.

Condition	Key Endothelial Mechanisms	Impact on Fracture Healing	Evidence Level	Principal Caveat	Key References
Diabetes mellitus	↓ Nitric oxide bioavailability, ↓ VEGF signaling, ↑ oxidative stress, ED	Delayed fracture healing, reduced angiogenesis, impaired callus formation	Human inception cohort, *n* = 47,437 patients/56,492 fractures (diabetes an independent nonunion predictor); mechanistic support from narrative reviews	Hyperglycemia, advanced glycation end products and neuropathy act in parallel; vascular contribution not isolated	[10,23]
Aging	↓ EPC number/function, ↓ angiogenic capacity, ↓ type H vessels	Reduced vascular density, delayed bone regeneration	Mouse, aged vs young, type H vessel quantification; human prospective study, *n* = 32 (16 aged < 40 y, 16 aged > 75 y), fracture hematoma and peripheral blood	Human angiogenic response was preserved in the elderly; endothelial effects inseparable from senescence, sarcopenia and frailty	[33,65,66,67]
Chronic kidney disease (CKD)	Uremia-associated ED, vascular calcification, ↓ NO bioavailability	Impaired remodeling, increased fracture risk, delayed healing	Rat, Cy/+ high-turnover CKD model, *n* = 6/group, endpoints 30 and 35 weeks; fluorescent-microsphere perfusion	Endpoint is skeletal perfusion, not union; marrow fibrosis of CKD–MBD operates largely independently of perfusion	[67,68]
Smoking	Endothelial injury, ↓ NO bioavailability, ↓ VEGF signaling, ↑ oxidative stress	Delayed union, nonunion risk, impaired vascularization	Rat, femoral osteotomy with cigarette-smoke inhalation, rat waterpipe smoke, femoral fracture; human inception cohort, *n* = 47,437	Independent endothelial contribution inseparable from nicotine toxicity, carbon monoxide–mediated hypoxia and direct osteoblast effects	[69,70]
Obesity	Chronic inflammation, ↑ oxidative stress, ↓ NO bioavailability, ED	Altered healing dynamics, impaired bone quality	Human inception cohort, *n* = 47,437; mechanisms characterized in general metabolic and cardiovascular populations, not fracture cohorts	Clinical evidence associative and inconsistent; ED is one plausible mediator among inflammatory, metabolic, osteogenic and mechanical pathways	[71]
Chronic Inflammatory Diseases	Persistent cytokine activation, endothelial activation, dysregulated angiogenesis	Impaired repair, inflammatory bone loss, disrupted remodeling	Mouse, serum-transfer-induced arthritis fracture model (nonunion, impaired angiogenesis); human cross-sectional RA cohort, RHI and pulse wave velocity vs BMD; human inception cohort, *n* = 47,437	Human vascular data address bone mineral density, not fracture repair; confounded by disease activity and glucocorticoid therapy	[72,73]

↑ increased; ↓ decreased.

**Table 2:** Key endothelial and angiogenic pathways relevant to fracture healing.

Pathway	Main Role	Fracture-Healing Relevance/Evidence Limitation	Citations
HIF-1α–VEGF	Links hypoxia to angiogenesis and vascular invasion	Strong experimental bone evidence; human studies are mainly observational and do not establish systemic ED as causal	[30,31,36,37]
Endothelial Notch	Regulates bone-vessel growth and osteogenic signaling	Mainly mouse developmental/homeostatic evidence; relevance to the adult human fracture callus remains unproven	[32,33,34]
Endothelial Noggin–BMP	Notch-regulated Noggin modulates BMP signaling and osteoprogenitor differentiation	Experimental evidence indicates that tightly regulated Noggin levels support bone formation; human fracture evidence is lacking	[32]
PDGF-BB/PDGF-AB	Supports vascular remodeling and angiogenic–osteogenic coupling	PDGF-BB is supported experimentally; lower circulating PDGF-AB has been observed in patients developing nonunion	[35,36]
EPCs	Contribute to vascular repair and neovascularization	Fracture-associated EPC changes have been reported, but EPC definitions and assays are heterogeneous; clinical therapeutic evidence remains preliminary	[40,41,46,47,48,49,50,51]

**Table 3:** Methods and biomarkers for assessing endothelial dysfunction.

Assessment	What It Reflects	Relevance to Fracture-Healing Research	Citations
Flow-mediated dilation (FMD)	Endothelium-dependent vasodilatory response	Established vascular research measure; potential research tool for prospective fracture cohorts	[24]
Nitroglycerin-mediated dilation (NID)	Endothelium-independent vasodilatory response	Helps distinguish endothelial dysfunction from impaired vascular smooth-muscle responsiveness	[24]
Reactive hyperaemia index (RHI)	Peripheral microvascular reactive hyperaemia	Complementary non-invasive vascular measure; not specific to fracture healing	[24]
VCAM-1/ICAM-1/E-selectin	Endothelial activation/inflammatory phenotype	Potential circulating markers of systemic vascular status	[25]
ADMA	Inhibitor of nitric oxide synthase activity	Candidate marker of impaired NO signaling	[25,52]
von Willebrand factor (vWF)	Endothelial activation/injury	Potential complementary marker of systemic endothelial disturbance	[25]
Circulating EPC measurements	Vascular repair/progenitor-cell reserve	Potential research biomarker, but interpretation is limited by EPC heterogeneity	[41,46,47,48,49,50]

**Table 4:** Glossary.

Term	Definition
ADMA	Asymmetric dimethylarginine; an endogenous inhibitor of NO synthesis and a marker associated with ED.
Angiocrine signaling	Paracrine signaling through which endothelial cells regulate neighboring osteogenic, progenitor and immune cells.
Angiogenic–osteogenic coupling	Coordinated interaction between new blood-vessel formation and bone formation during skeletal repair.
ED	Endothelial dysfunction; impaired endothelial regulation characterized by reduced NO bioavailability, oxidative stress and inflammatory or pro-thrombotic activation.
eNOS/NO	Endothelial NO synthase/nitric oxide; a major pathway regulating vascular tone, perfusion and endothelial homeostasis.
EPCs	Endothelial progenitor cells; heterogeneous circulating cell populations used as indirect indicators of vascular repair capacity.
FMD, NID and RHI	Flow-mediated dilation, nitroglycerin-mediated dilation and reactive hyperaemia index; functional methods used to assess vascular or endothelial responses.
HIF-1α–VEGF axis	Hypoxia-responsive pathway that promotes endothelial recruitment, angiogenesis and vascular invasion of the fracture callus.
PDGF-BB	Platelet-derived growth factor-BB; a growth factor involved in vascular remodeling, endothelial recruitment and angiogenic–osteogenic coupling.
Type H vessels	Specialized bone capillaries characterized by high CD31 and endomucin expression and closely associated with osteoprogenitor cells and bone formation.

## Data Availability

No new data were created or analyzed in this study. Data sharing is not applicable to this article.
